# Early-Life Stress Influences the Transcriptional Activation of Alpha-2A Adrenergic Receptor and Associated Protein Kinase A Signaling Molecules in the Frontal Cortex of Rats

**DOI:** 10.1007/s12035-024-04578-7

**Published:** 2024-11-13

**Authors:** Sarah Ali, Yogesh Dwivedi

**Affiliations:** Department of Psychiatry and Behavioral Neurobiology, University of Alabama at Birmingham, SC711 Sparks Center, 1720 2nd Avenue South, Birmingham, AL USA

**Keywords:** Early-life stress, Alpha-2A adrenergic receptor, Protein kinase A, Rat frontal cortex, Signaling system

## Abstract

Early life is a highly sensitive period associated with profound changes in brain structure and function. Adverse experiences of early-life stress (ELS) are prominent risk factors for the precipitation of major depressive disorder (MDD). In recent years, dysfunction of the central noradrenergic (NA) system and subsequent deficits in norepinephrine (NE) signaling have gained increasing attention in the pathophysiology of MDD. However, the role of the α-2A adrenergic receptor and its downstream second messenger signaling system has not been investigated in connection to early-life stress-induced depression, limiting valuable insights into neurobiological mechanisms underlying this disorder. In this study, we used maternal separation (MS) as a rodent model of ELS to investigate whether ELS-induced depressive behavior is related to the α-2A adrenergic receptor and its associated second messenger signaling cascade. To do so, we studied expression levels of the α-2A adrenergic receptor (*Adra2a*), G alpha proteins (stimulatory subunit-G_αs_ [*Gnas*] and inhibitory subunit-G_αi_ [*Gnai1* and *Gnai2*]), and downstream protein kinase A (PKA) catalytic [*Prkarc*α and *Prkarcβ*] and regulatory subunits [*Prkar1α*,* Prkar1β*,* Prkar2α*, and *Prkar2β*]) in the frontal cortex (FC) of MS rats. We found reduced sucrose preference in MS animals, along with reduced transcript levels of *Adra2a*, *Gnai2*, *Prkar1β*, and *Prkarcβ*. These findings suggest that ELS exposure may contribute to depression symptomatology via alterations in the expression of key genes involved in the NA system, highlighting potential mechanisms underlying ELS-induced depressive behavior.

## Introduction

Early life is a highly vulnerable and critical period for cognitive development, synaptic plasticity, and the formation of underlying neural circuitry in the central nervous system (CNS) [1–6]. Adverse experiences of early-life stress (ELS) during this period, including neglect, physical, emotional, and sexual abuse, social deprivation, and household dysfunction, have been shown to exert a profound impact on these processes [7, 8]. In the United States, approximately 600,000 cases of child abuse and neglect were confirmed in 2021 [9]. Such adverse experiences are prevalent and long-lasting, with epidemiological data consistently linking ELS to negative health outcomes in adult life, including physical illnesses such as cardiovascular disease, cancer, diabetes, stroke, and respiratory disease [7, 10–15]. Exposure to ELS is also a prominent risk factor for the precipitation of various psychopathologies, including major depressive disorder (MDD), bipolar disorder, posttraumatic stress disorder (PTSD), schizophrenia, and suicidal behavior [10, 16–21]. While MDD is patient-specific, its onset and progression are primarily driven by stress. MDD is one of the most burdensome illnesses globally, with a lifetime prevalence rate of 21% [22]. Despite increasing rates of antidepressant use over time, only around 50% of MDD patients respond to the existing medical therapy, while a substantial proportion of MDD patients suffer from treatment-resistant depression [23–25]. Although our understanding of the neurobiological basis of MDD has improved, the etiology of MDD and its effective treatment strategies remain elusive.

Several lines of evidence suggest that there is a strong correlation between ELS and depression symptomatology. For example, recurring experiences of ELS compared to those who experienced none had an increased risk for MDD by 4-fold [10]. Additional meta-analysis investigating the association between depression and experiences of childhood adversity revealed that maltreated individuals were twice as likely to develop multiple, persistent depressive episodes in adulthood, had an earlier onset of MDD, and were twice as likely to develop treatment-resistant depression [26]. These poor outcomes emphasize the critical need to investigate the biological mechanisms triggered by ELS that precipitate MDD.

Dysfunction of the central noradrenergic (NA) system and subsequent deficits in norepinephrine (NE) signaling have been implicated in the pathophysiology and treatment of MDD [27–29]. Specifically, the α-2A adrenergic receptor (*Adra2a*) and associated adenylyl cyclase (AC)-cAMP activity have been shown to modulate poor executive function and neuroinflammation in depression [30, 31]. The α-2A adrenergic receptor is a subtype of G-protein-coupled receptors (GPCRs) that couples with the inhibitory subunit of G alpha protein (G_αi_) to suppress intracellular cAMP levels [27, 31]. Further, protein kinase A (PKA), a crucial downstream effector molecule in the cAMP signaling pathway, transduces extracellular signals through the phosphorylation of target proteins to regulate gene expression, neuronal plasticity, and responses to stress [32–34]. Previous studies have reported PKA regulatory and catalytic subunit dysfunction as well as decreased levels of cAMP in the frontal cortex of depressed subjects who died by suicide [35, 36]. This accumulating evidence emphasizes the involvement of aberrant PKA activity in depression pathophysiology. However, the role of *Adra2a* and its related signaling molecules, such as cAMP and PKA, remains largely unexplored in the context of ELS-mediated MDD. This lack of understanding poses a significant barrier to gaining a deeper understanding of the adrenergic neurobiology underlying depression susceptibility and effective treatment options that can improve patient outcomes.

Using a rodent model of ELS (maternal separation; MS), the present study aims to investigate whether ELS-induced depressive behavior can be linked to the α-2A adrenergic receptor and its associated signaling pathway. For this, we studied the expression of *Adra2a*, G alpha protein stimulatory-G_αs_ and inhibitory-G_αi_ subunits, as well as catalytic and regulatory subunits of PKA in the frontal cortex (FC). It was hypothesized that exposure to early-life stress alters the expression of *Adra2a*, affecting subsequent noradrenergic neurotransmission and dysregulating the linked downstream effectors of the AC-cAMP signaling pathway.

## Materials and Methods

All experiments were performed in accordance with the National Institutes of Health (NIH) guide for the care and use of Laboratory animals and were approved by the University of Alabama at Birmingham’s Animal Care Committee (IACUC).

### Animals

**Fig. 1:**
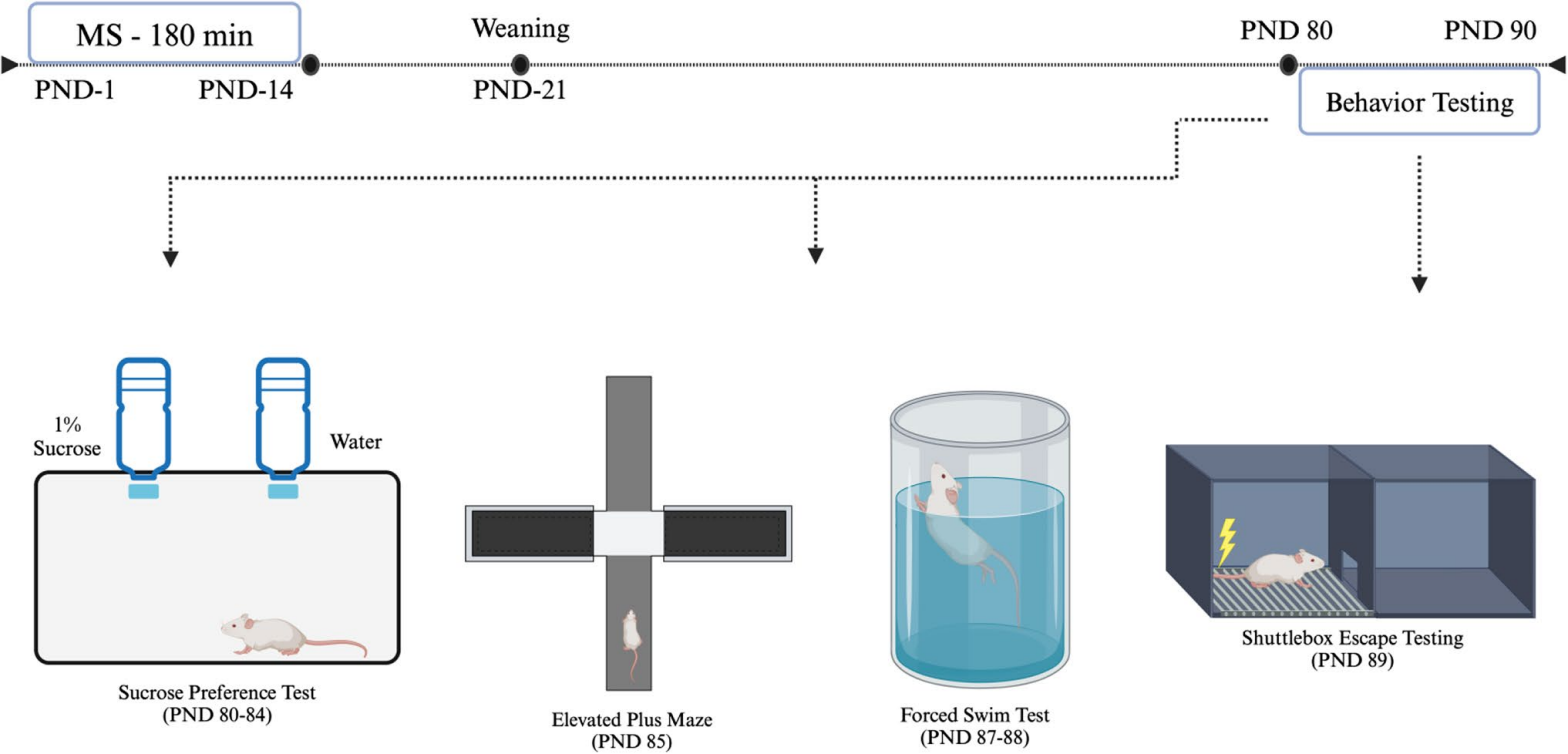
Schematic timeline summarizing the experimental methodology. Female, pregnant Holtzman rats housed under standard laboratory conditions were accustomed to their environment for one week prior to the start of experiments. Pups were born (PND 0) and randomly divided into the control and MS groups. Animals in the control group were handled for five minutes daily from PND 1–14 while animals in the MS group were separated from the dam for 180 min per day from PND 1–14. The MS rats underwent behavior testing via sucrose preference test (PND 80–84), EPM (PND 85), FST (PND 87–88), and shuttle box escape test (PND 89). MS = maternal separation; PND = postnatal day; EPM = elevated plus maze; FST = forced swim test

Female, pregnant Holtzman rats were obtained from Envigo (Indianapolis, IN, USA) and were housed under standard laboratory conditions (temperature 21 ± 1 ºC, humidity 55 ± 5%, 12-hour light/dark cycle). Animals were given unlimited access to food and water and acclimated to the laboratory environment for one week before the experiments began. Dams were monitored twice daily from gestational day 20 until pups were born (postnatal day [PND] 0). After the birth of the pups, the litter was randomly divided into two groups: the control group and the maternal separation (MS) group. Pups in the control group were handled for 5 min each day from postnatal day (PND) 1–14. Pups in the MS group were separated from the dam and housed individually for 180 min (3 h) daily from PND 1–14. Earlier, our lab reported depressive phenotypes in these MS rats via behavioral testing, including sucrose preference, elevated plus maze, forced swim test, and shuttle box escape testing [37]. Each rat was tested for depression- and anxiety-related behaviors as follows: sucrose preference test on PND 80–84, elevated plus maze (EPM) on PND 85, forced swim test (FST) on PND 87–88, and shuttle box escape test on PND 89. A timeline detailing the experimental design is shown in Fig. 1. For this current study, 6 handled-control rats, along with 6 male MS rats, were analyzed.

### Behavior Testing

#### Sucrose Preference Test

Rats were assessed for deficits in response to pleasure and motivation, specifically anhedonia, via the sucrose preference test [38]. Initially, the rats were accustomed to two 500-mL bottles of 1% sucrose water for 24 h, followed by one bottle of sucrose water and one bottle of tap water for each rat. Food and water were then withheld for 24 h. Subsequently, each rat was individually housed with access to food and two tightly sealed 500-mL bottles, one containing tap water and the other with 1% sucrose water. Following an additional 24 h, the volume of liquid consumption was measured. Sucrose preference was calculated as the percentage of sucrose water consumed relative to the total liquid intake using the following formula: (mL of sucrose solution / [mL of sucrose solution + mL of tap water]) x 100.

#### Elevated Plus Maze

Following a 30-minute adjustment period in the testing room, each rat was individually positioned at the center of a 100-cm elevated plus maze (EPM). The movements of the rats were monitored for 5 min using EthoVision XT 11.5 (Noldus) as mentioned previously [37]. An anxiety index considering the number of entries into the open arms, the total number of arm entries, and the overall test duration was calculated.

#### Forced Swim Test

24 h prior to testing, each animal was accustomed to a Plexiglas cylinder (~ 30 cm in width x ~ 40 cm in length) filled with ~ 20 cm of room temperature water for 15 min. During the testing phase, the subjects swam under identical conditions each day for 6 min as performed previously [37]. The entirety of the session was video recorded and analyzed using Kinoscope [39] software to derive swim, climb, and immobility scores.

#### Shuttle Box Escape Testing

Twenty-four hours after the forced swim test, escape testing was conducted in a shuttle box (70 × 20 × 20 cm) equipped with an electrified grid floor. A foot shock was administered via the grid floor using a shock generator set to 0.6 mA on a variable interval schedule as described earlier [37]. The testing protocol started with five trials in which a single crossing eliminated the shocks, then proceeded with 25 trials where the animal crossed and returned to the original position to terminate the shocks. The shocks ceased after 30 s, and the escape latency was measured.

#### Brain Tissue Collection

At 24 h following escape testing, rats were sacrificed in adulthood (PND 90). Adrenal glands from each animal were collected and weighed. Rat brains were dissected and flash-frozen with liquid nitrogen. A serial dissection using a cutting block was performed following the reported protocol [40]. The FC included the frontal poles as well as cortical tissue superior to the rhinal sulcus. Only tissue superior and rostral to the corpus callosum was collected and stored at -80 ºC until analysis.

#### mRNA Primer Design

Primers for the coding gene were designed using rat-specific sequences sourced from the Rat Genomic Database (RGB). The primers were proximal to the 3’ end of the transcript to ensure efficacy. The specificity of the primer sequences was assessed via a BLAST search against the NCBI nucleotide (NT) database and optimized accordingly to eliminate potential off-target amplification. The primers used in the present expression study are detailed in Table 1.

**Table 1:** mRNA oligo sequences for qPCR-based gene expression analysis

Gene	Forward (5’-3’)	Reverse (5’-3’)
*Gapdh*	ATT CCA TCC CAG ACC CCA TAA C	GTG CAG CGA ACT TTA TTG ATG GTA T
*Adra2a*	AGC TCG CTG AAC CCT GTT AT	CTG ACC AGG GTC TGT AAG CA
*Gnas*	GGT GAG AAG GCC ACC AAA GT	AAC TGG TTC TCA GGG TTG GC
*Gnai1*	TTG GTT CTG TGT TTG GCA GTT	CAG GGT AAG GGG GTT GAC ATT
*Gnai2*	TCC CTG TCT AAA ACC CAC CTT	AGA AAA CCC GAA TGG ATG CC
*Prkar1 *${\alpha}$	GCG TCG GTC AGA AAA CGA AG	ACG ATT CAT CAG GGC AA
*Prkar1* ${\beta}$	AAC CTG CCT ATT GGA GAC CC	GAG CAG AGA GGT TTG GGA GT
*Prkar2* ${\alpha}$	CCG GGC AGT AGA TGT GAT GAA	GGT GTG TTC TTG TGG CTG AC
*Prkar2* ${\beta}$	CTG GCT CAT CCT TCT GTG TTC T	TCC ACA GGC ATT GGT TTC CG
*Prkarc* ${\alpha}$	CGT GTG AAA GGC CGA ACT TG	AAC CAG CCA TCT CGT AG
*Prkarc* ${\beta}$	GGG TTC GCC AAG AGA GTC AA	TAG CCA GCA GCC ATC TCG TA

#### Rat Brain RNA Isolation and First Strand cDNA Synthesis

Total RNA was isolated using TRIzol reagent (Invitrogen Life Technologies, USA) as outlined previously [41]. Following phase separation with chloroform, the aqueous phase was obtained, and the RNA was precipitated using isopropanol and 20 µg of glycogen. The reaction was allowed to proceed overnight at − 30 ºC. Afterward, the RNA pellet was washed with 70% ethanol, and the pellet was dried before resuspension in nuclease-free water. The RNA samples were characterized for purity (260/280 absorbance ratio; cutoff ≥ 1.8) as determined with NanoDrop spectrophotometer (Thermo Scientific, Waltham, MA). The RNA quality was further assessed based on agarose gel electrophoresis.

Complementary DNA (cDNA) was synthesized using 1 µg of total isolated RNA following an oligo dT-based priming method aided by reverse transcription with M-MLV Reverse Transcriptase (Invitrogen, NY, USA) as detailed previously [41]. The oligo dT-primer annealing stage was performed with a concentration of 5 μm alongside 1mM of dNTPs. The reaction was first incubated at 65ºC for 5 min and then inactivated at 4ºC for 2 min. The reverse transcription step was conducted by adding 1X first strand synthesis buffer, 0.01mM DTT, 2 U of RNaseOut, and 200 U of M-MLV Reverse Transcriptase. The reaction underwent incubation for 50 min at 37ºC before being further inactivated at 70ºC for 15 min.

#### qPCR-Based mRNA Expression Assay in Rat FC

Relative transcript abundance of mRNA coding genes was tested by real-time qPCR in combination with 1X EvaGreen qPCR master mix (Applied Biological Material, Richmond, Canada). Genes with direct involvement of the α-2A adrenergic receptor and its signal transduction pathway, including the inhibitory-G_αi_ and stimulatory-G_αs_ protein subunits, as well as the catalytic and regulatory PKA subunits, were selected for expression analyses. Gene-specific forward and reverse primers were evaluated at a concentration of 0.5 µM.

Raw cDNA was diluted forty-fold and utilized as a template for qPCR amplification. The thermal parameters for qPCR amplification comprised of an initial denaturation at 95 °C for 10 min, followed by 40 consecutive cycles of denaturation at 95 °C for 10 s, primer annealing at 60 °C for 15 s, and an extension step at 72 °C for 20 s. The possibility of dimerization and amplification of unintended products was effectively prevented via qPCR analysis of non-template samples. Relative gene expression levels were normalized with *Gapdh*, and fold-change was determined with Livak’s ΔΔCT method [42].

### Statistical Analysis

The data was analyzed using a Student’s T-test to evaluate the effects of ELS on the rats and identify any significant impacts resulting from the stress paradigm. For the gene expression assays, the transcription levels of *Gapdh*, the normalizer, did not differ significantly between the groups. The results are expressed as the mean ± SEM, with the significance level *p* ≤ 0.05. The Levene’s Test for Equality of Variances was also performed to assess the homogeneity of variances across the groups and experimental conditions by the SPSS statistical package (IL, USA).

## Results

### Animal Behavior

**Fig. 2:**
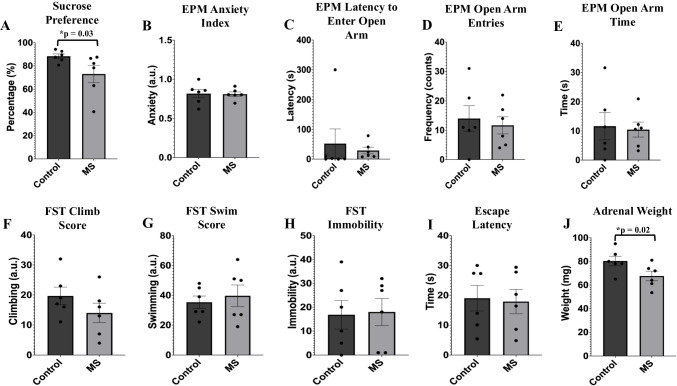
Behavioral changes observed following assessments of control and MS animals. The mean behavior differences are shown as dot plots. Data are mean ± SEM. **A** Sucrose preference was significantly reduced following MS (*p* = 0.03). **B** Animals of the MS group showed no variations in anxiety-like behaviors following EPM (*p* = 0.45). The anxiety index derived from the EPM incorporates measures including the latency to enter open arms, number of open arm entries, and time spent in the open arms. **C** The latency to enter open arms of the EPM showed no significant changes between both groups despite the reduced latency shown for MS animals (*p* = 0.3). **D** The number of open arm entries derived to calculate the anxiety-related value showed non-significant differences between MS animals and handled-control animals (*p* = 0.3). **E** The time spent by the animals in the open arms of the EPM showed no significant variations following MS in comparison to the handled-control group (*p* = 0.4). **F** Although not statistically significant, FST climb scores were reduced in MS animals during forced swim test (*p* = 0.11). **G** and **H** There were no significant changes for either FST swim scores or FST immobility scores between the two groups of animals (*p* = 0.3 and *p* = 0.4, respectively). **I** MS animals did not show significant variations in escape latency after shuttle box escape testing (*p* = 0.4). **J** The MS group did show a reduction in adrenal weight at a significant level (*p* = 0.02) in comparison to the handled-control group. Significance was determined using Student’s T-test (*n* = 6 per group, *N* = 12); *p* ≤ 0.05. MS = maternal separation; a.u. = arbitrary units; EPM = elevated plus maze; FST = forced swim test

Student’s T-test showed a significant effect of sucrose preference, revealing significantly decreased preference (*p *= 0.03, Fig. 2A) in the MS group compared with the handled-control animals. Contrarily, the anxiety index, which combines various EPM measures into a single numerical value, showed no differences between both the control group and the MS group and failed to reach a value of significance (*p* = 0.45, Fig. 2B). No significant differences were also found in the latency of the animals to enter the open arm of the EPM (*p* = 0.3), the number of entries into the open arms of the EPM (*p* = 0.3), or the time spent in the open arms of the EPM (*p* = 0.4) across both handled-control and MS animals (Fig. 2C, 2D, and 2E, respectively). For the forced swim test, climb scores decreased in the MS group but not significantly (*p* = 0.11, Fig. 2F). The additional forced swim test scores, including swim (*p* = 0.3) and immobility (*p* = 0.4), did not show significant differences between the male MS rats and the handled-control rats (Fig. 2G and H). Additionally, according to the escape latency measured from the shuttle box escape test, no significant differences were found following MS (*p* = 0.4, Fig. 2I).

### Adrenal Weight Analysis

A significant decrease in adrenal weight (*p* = 0.02) was found between the handled-control group and the MS group, with adrenal weight decreasing after MS in the male animals (Fig. 2J).

### mRNA Expression Analysis

Gene expression analysis was conducted in behaviorally tested brain regions (FC) of handled-control and MS rats to determine the effects of early life stress exposure on genes associated with the α-2A adrenergic receptor and its downstream targets associated with the AC-cAMP signaling pathway. When assessing the variance within the calculated ratios, one handled-control rat sample was excluded from all analyses due to exhibiting high variance. The mRNA expression data for all genes is represented as dot plots as well as bar diagrams. The dot plot includes the normalized ΔCT (dCT) values for each individual sample, with the bars indicating the average ΔCT values. The individual ΔCT values for each sample were averaged within the handled-control and MS group to obtain the mean ΔCT for each group. The ΔΔCT (ddCT) was then calculated by subtracting the control group’s average ΔCT from that of the MS group. This ΔΔCT value reflects the relative difference in mRNA expression between the two groups, normalized to the reference gene. The ΔΔCT was used to calculate the fold change in gene expression by applying the following formula: 2^-ΔΔCT. Fold change translates this difference into a more intuitive ratio that reflects how many times gene expression has increased or decreased between the groups. This fold change quantifies the magnitude of change in transcript expression between the MS and the handled-control groups. The relative fold-change for each target gene is illustrated via bar diagrams.

### Expression of α-2A Adrenergic Receptor

**Fig. 3:**
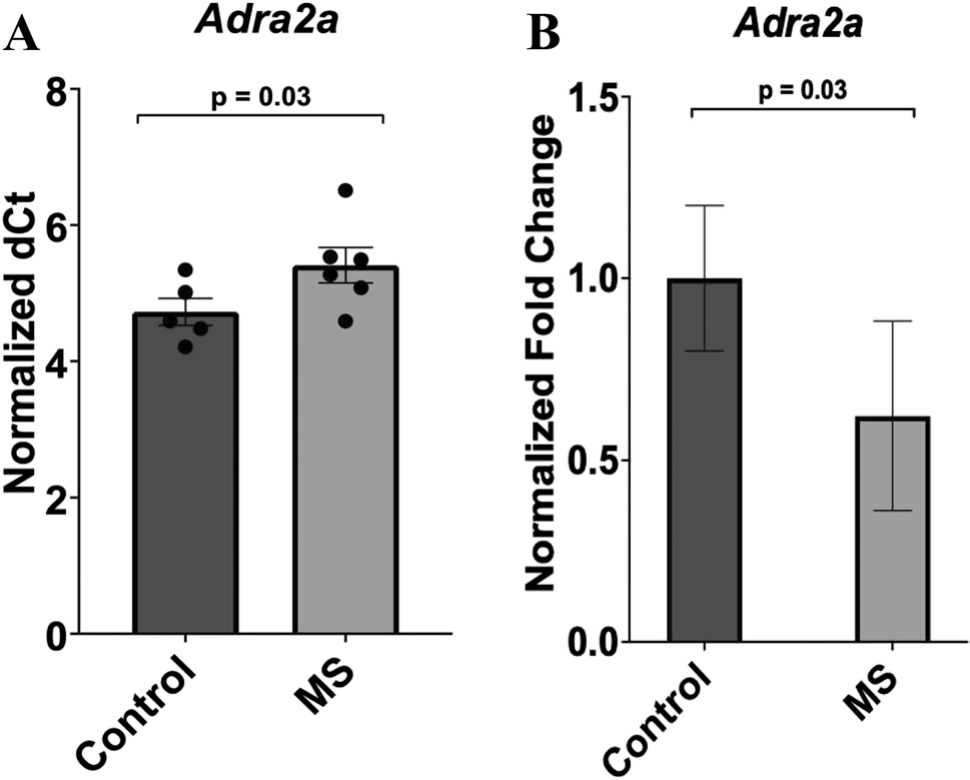
Expression of the a-2A adrenergic receptor in the frontal cortex of handled-control and MS rats. The differences in relative transcript expression, shown as fold change in the bar plots, was calculated by Student’s T-test and the SPSS statistical package. Data are the mean ± SEM. **A** The normalized ΔCT values for individual samples, along with the average ΔCT for the handled-control and MS groups, are shown in the dot plot. The mean ΔCT values for each group were used to calculate the ΔΔCT, which was then applied to the formula 2^-ΔΔCT to determine the fold change. **B** Significant differences were found in the MS group (*n* = 6) compared to the handled-control animals (*n* = 5), with MS animals showing significantly decreased expression of *Adra2a* (*p* = 0.03, F = 0.120, t=-2.018, f = 9). Data was normalized against *Gapdh*. MS = maternal separation; *Adra2a* = a-2A adrenergic receptor

RNA expression for the α-2A receptor (*Adra2a*) in the FC of MS rats showed significant expression changes between the handled-control group and the MS group (*p* = 0.03, F = 0.120, t=-2.018, df = 9). The mRNA expression data for *Adra2a* is represented as dot plots and bar diagrams. The individual and average ΔCT values for both groups are shown in the dot plot (Fig. 3A**)**. Using these values to calculate the ΔΔCT and applying the fold change formula, the MS group showed significantly reduced mRNA transcript levels by approximately 38% compared to the handled-control group, with a relative fold change decrease from 1.0 to 0.62, as depicted in the bar plot (Fig. 3B**).**

###  Expression of G Alpha Protein Inhibitory (Gαi) and Stimulatory (Gαs) Subunits

**Fig. 4:**
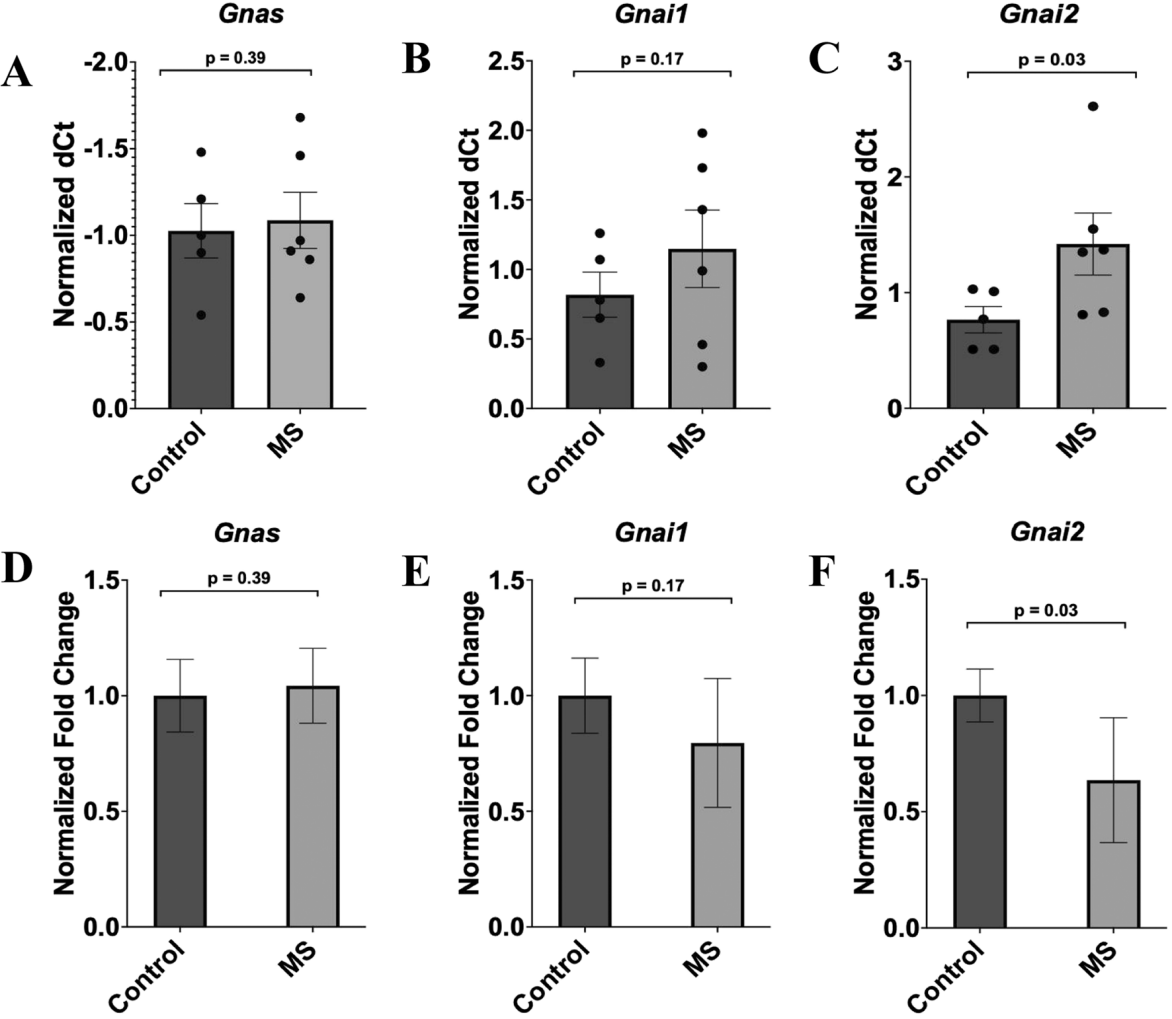
Expression of G alpha inhibitory and stimulatory subunits in the frontal cortex of MS animals compared to handled-control animals. Using Student’s T-test and the SPSS statistical package, fold change was calculated and is shown via the bar diagrams. Data are the mean ± SEM. The stimulatory subunit-G*a*s is *Gnas*. The inhibitory subunits-G*a*i are *Gnai1* and *Gnai2*. The normalized ΔCT values as well as the mean ΔCT for each group are depicted via the dot plots (**A**, **B**, **C**). These values were used to find the ΔΔCT values, and calculate the fold change using the following formula: 2^-ΔΔCT. **D** No significant differences in mRNA expression were found between both the handled-control (*n* = 5) and MS group (*n* = 6) for *Gnas* (*p* = 0.39, F = 0.333, t = 0.266, df = 9). **E** *Gnai1* showed reduced expression levels in the MS group (*n* = 6) but did not reach significance (*p* = 0.17, F = 3.660, t=-0.969, df = 9). **F** *Gnai2* showed significantly reduced expression following MS (*p* = 0.03, F = 1.294, t=-2.082, df = 9). Data was normalized against *Gapdh*. MS = maternal separation

The individual ΔCT values for each sample and the mean ΔCT values for each group are given for both G alpha inhibitory and stimulatory subunits as dot (Fig. 4A–C) and bar (Fig. 4D–F **)** plots, respectively, providing additional context for the calculation of the fold change. The mRNA analysis showed that the stimulatory subunit-Gαs, *Gnas*, had no changes in relative transcript levels between the handled-control and MS group (*p* = 0.39, F = 0.333, t = 0.266, df = 9; Fig. 4D), with the fold change for the MS group remaining around 1.0. On the other hand, significant expression differences were noted between the handled-control and MS group for the G alpha protein inhibitory subunit-Gαi, *Gnai2*. The MS rats showed significantly downregulated expression (*p* = 0.03, F = 1.294, t=-2.082, df = 9; Fig. 4F), reflecting a reduction in expression by 36% from 1.0 to 0.64 when compared against the handled-control group. The other inhibitory G alpha protein subunit-Gαi, *Gnai1*, showed reduced mRNA levels in the MS rats by 21% with a fold change decrease from 1.0 to 0.79, but did not reach a significant level (*p* = 0.17, F = 3.660, t=-0.969, df = 9; Fig. 4E).

### Expression of PKA Regulatory and Catalytic Subunits

**Fig. 5:**
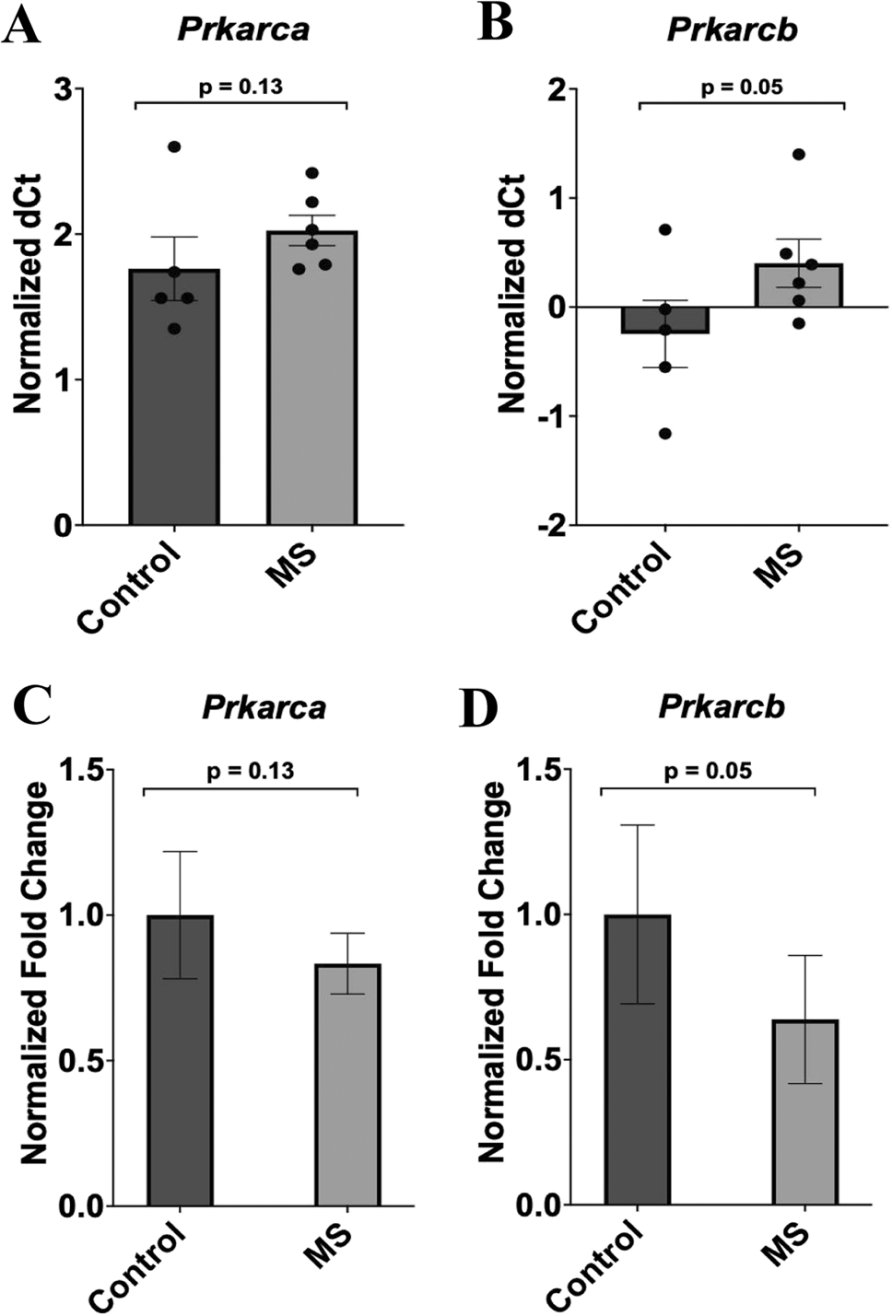
Expression of PKA catalytic subunits in the frontal cortex of handled-control and MS rats. The changes in transcript expression levels are shown as fold change in the bar plots, which was calculated by Student’s T-test and the SPSS statistical package. Data are the mean ± SEM. The normalized ΔCT values for each individual sample, in addition to the mean ΔCT for the handled-control and MS groups are provided in the dot plots (**A**, **B**). These data were used to find the ΔΔCT values, and the fold change was found using the formula 2^-ΔΔCT. The two catalytic subunits are Prkarc*a* and Prkarc*β*. **C** *Prkarca* showed reduced mRNA transcript levels in the MS animals (*n* = 6), but the results were not significant (*p* = 0.13, F = 0.948, t=-1.151, df = 9). **D ***Prkarcβ* did show significant results with downregulation in the MS group (*n* = 6) in comparison to the handled-control animals (*n* = 5, *p* = 0.05, F = 0.278, t=-1.752, df = 9). Data was normalized against *Gapdh*. MS = maternal separation

**Fig. 6:**
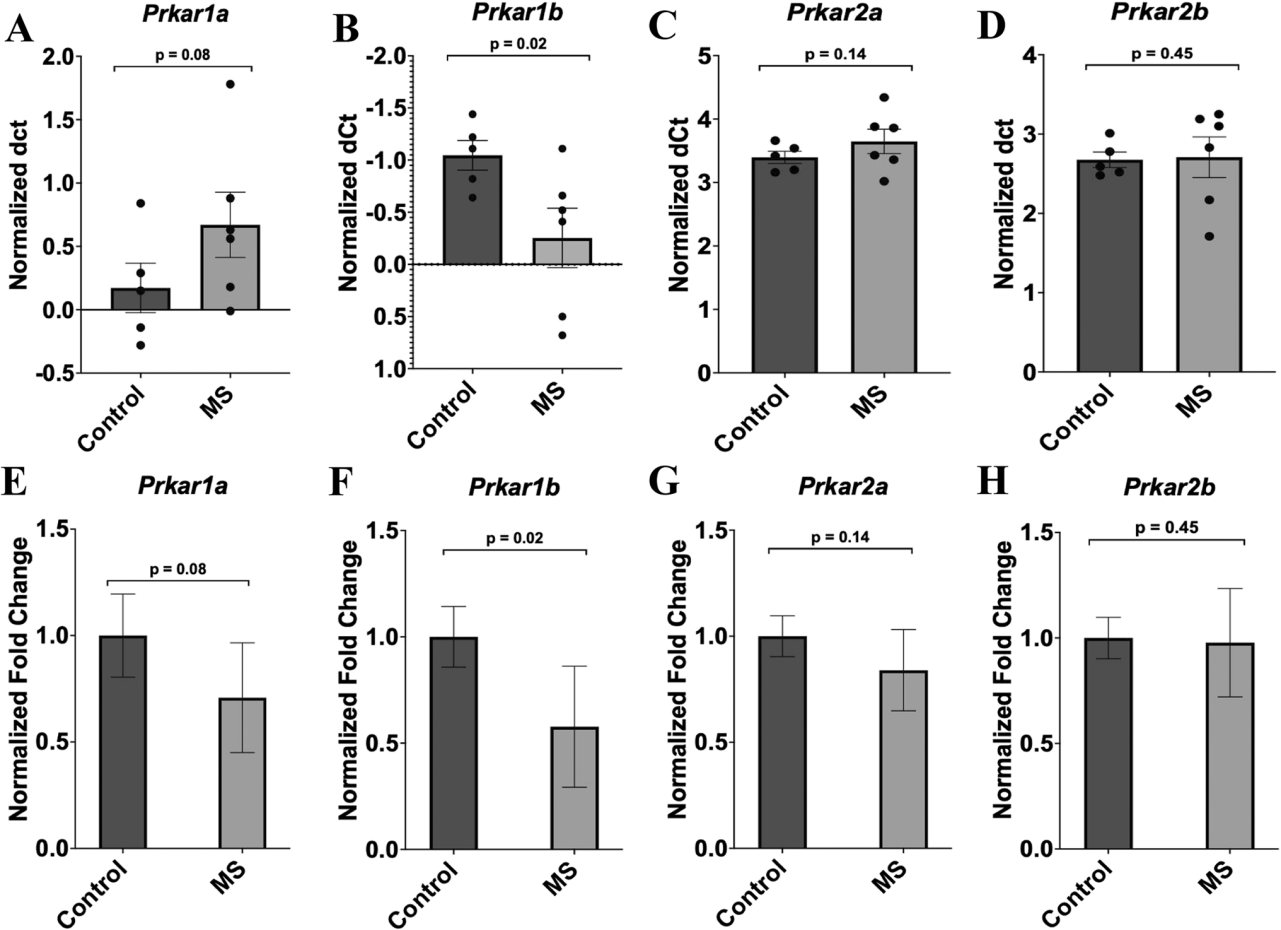
Expression of regulatory PKA subunits in the frontal cortex of handled-control and MS rats. Gene expression differences are shown as fold change in the bar diagrams, which was found using Student’s T-test and the SPSS statistical package. Data are the mean ± SEM. Normalized ΔCT values for each animal and the average ΔCT value for each group is shown in the dot plots (**A**, **B**, **C**, **D**). These ΔCT values were used to derive the ΔΔCT values, which were then applied to determine the fold change via the formula 2^-ΔΔCT. The four regulatory subunits are Prkar1*a*, Prkar1*β*, Prkar2*a*, and Prkar2*β*. **E** *Prkar1a* showed decreased expression between the two groups but was higher than the necessary significance value (*p* = 0.08, F = 0.352, t=-1.487, df = 9). **F ***Prkar1β* did show significant results of downregulated mRNA transcript levels following MS (*p* = 0.02, F = 3.772, t=-2.331, df = 9). **G ***Prkar2a* showed reduced expression within the MS group (*n* = 6), but the significance value was *p* > 0.05 (*p* = 0.14, F = 3.710, t=-1.102, df = 9). **H** The remaining subunit, Prkar2*β*, showed no variations in expression between both groups and the results were also not significant (*p* = 0.45, F = 6.267, t=-0.109, df = 9). Data was normalized against *Gapdh*. MS = maternal separation

The average ΔCT values for both the MS and handled-control groups, as well as the individual ΔCT values from which these averages were derived, are provided as dot plots for catalytic (Fig. 5A and B**)** and regulatory (Fig. 6A–D) subunits. These data were used to determine the ΔΔCT and the resulting fold change difference between the groups. Of the two catalytic PKA subunits, *Prkarcα* showed downregulated expression in the MS group by approximately 17% with a fold change of 1.0 to 0.83; however, the expression changes failed to reach the significance cutoff of *p* ≤ 0.05 (*p* = 0.13, F = 0.948, t=-1.151, df = 9; Fig. 5C). On the other hand, *Prkarcβ* showed significantly reduced expression (*p* = 0.05, F = 0.278, t=-1.752, df = 9) by 36% in the MS group compared to the handled-control group, with a fold change decrease from 1.0 to 0.64, as depicted in Fig. 5D. The four regulatory PKA subunits also showed interesting expression changes. *Prkar1α* was downregulated in the MS rats by 30% as the fold change was 0.70, but marginally failed to reach the significance value (*p* = 0.08, F = 0.352, t=-1.487, df = 9; Fig. 6E). Further, in comparison to the control group, the MS group did show significantly decreased expression for the *Prkar1β* subunit (*p* = 0.02, F = 3.772, t=-2.331, df = 9; Fig. 6F) by over 40% with a fold change reduction to 0.57 in the MS group. For the remaining regulatory subunits, *Prkar2α* and *Prkar2β*, the MS group exhibited decreased levels of *Prkar2α* by 16% with a fold change of 0.84, and no change in transcript expression was observed for *Prkar2β*, as the fold change for the MS group remained similar to that of the handled-control group. Neither *Prkar2α* nor *Prkar2β* reached the statistically significant level (*Prkar2α*: *p* = 0.14, F = 3.710, t=-1.102, df = 9; *Prkar2β*: *p* = 0.45, F = 6.267, t=-0.109, df = 9; Fig. 6G and H).

## Discussion

The present study aimed to understand ELS-induced major depression and its association with the α-2A adrenergic receptor signaling cascade in a rodent model of maternal separation. Our behavioral data demonstrated that MS animals failed to show changes in the forced swim score (an indicator of despair behavior), escape latency (an indicator of learned helplessness), or EPM latency (an indicator of anxiety-like behavior); however, they exhibited significantly decreased sucrose preference, demonstrating an anhedonic state, a primary indicator of depression-like behavior. MS animals also showed lower adrenal weight, showing a sign of adrenal insufficiency. When examining the qPCR-based expression of the alpha-adrenergic receptor, *Adra2a*, we found significantly reduced expression of this NA receptor in the FC of MS rats. Additionally, the expression of *Gnai2* was decreased in the FC of MS rats, along with decreased expression of *Prkar1β* and *Prkarcβ*. Several other genes associated with G proteins and PKA regulatory and catalytic subunits, such as *Gnai1*, *Prkar1α*, *Prkar2α*, and *Prkarcα* indicated lower expression; however, they did not reach statistical significance. Similarly, no significant differences in the expression levels of *Gnas* and *Prkar2β* were observed in the MS rats compared to handled-control rats. Altogether, our data, for the first time, demonstrates that early-life stress-induced depressive behavior could be associated with abnormalities in the α-2A adrenergic receptor and its linked signal transduction mechanisms. Our study is consistent with previously reported findings showing reduced expression of *Adra2a* [43], G alpha protein inhibitory subunit-G_αi_ [44, 45], and PKA regulatory and catalytic subunits [35, 36] in depressed patients, although these studies were not done in the context of early-life stress. It is interesting to note that only a few studies have demonstrated lower α-2A adrenergic receptor density, whereas consistent lines of evidence have shown upregulation in the frontal cortex, hippocampus, and hypothalamus of depressed patients and suicide subjects [46–48].

The NA system holds significant relevance in the context of depression, exerting effects on its manifestation and symptom severity [27, 49]. The NA system is mainly located in the locus coeruleus of the brainstem, the primary source of central norepinephrine (NE) synthesis; however, noradrenergic projections reach areas throughout the brain, including the hypothalamus, limbic regions, thalamus, cortex, and olfactory bulb. The FC is one of the major areas innervated by noradrenergic neurons. The action of NE is mediated by the family of G protein-coupled receptors known as the adrenergic receptors. Among the various adrenergic receptors, α-2A adrenergic receptors are the most important receptor family involved in the regulation of noradrenergic neurotransmission and thus have been subject to intensive investigation for potential roles in depression. The α-2A adrenergic receptor family consists of α_2A_, α_2B_, and α_2C_ receptors encoded by independent genes [30, 50]. Among them, the α-2A adrenergic receptor is predominantly expressed in the brain [51, 52] and is largely responsible for central noradrenergic activities. Whereas the α-2B adrenergic receptor is expressed in peripheral tissues, the α-2C adrenergic receptor has a distinct mechanism of action on noradrenergic activity. α-2A adrenergic receptors are coupled with the G_αi_ subunits to inhibit AC activity and consequent cAMP formation. In the present study, as indicated above, we found that the expression of *Adra2a* was significantly decreased in the FC of MS rats. This is contrary to what has been previously reported in human postmortem brain studies of depressed and suicidal individuals. These studies suggest that agonist binding to α-2A adrenergic receptors is either unchanged or elevated in various projection regions in depression and suicide [53]. An mRNA expression study also shows that its level is increased in the frontal cortex of suicide subjects, most of them having depression diagnoses [46]. Further, a significant increase in α-2A adrenergic receptor immunoreactivity has been shown in the FC of depressed subjects who died by suicide [54]. It has been hypothesized that supersensitive presynaptic α_2_-adrenoceptors could reduce NE release in depression. Alternatively, based on animal model studies, it has been hypothesized that the pharmacological depletion of NE can result in an adaptive upregulation of α_2_-adrenoceptors [55–57]. In this regard, our study of ELS-induced depression in rodents is opposite to these findings. Since *Adra2a* is an inhibitory presynaptic receptor, decreased *Adra2a* expression indicates increased NE activity in MS rats [27]. Although speculative in nature, the observed downregulation of *Adra2a* could be due to reduced feedback inhibition within the noradrenergic system. With fewer inhibitory receptors available, it is possible that the regulatory control over NE release and signaling may be impaired, leading to aberrant processing of downstream signaling and postsynaptic adrenergic receptors, contributing to neurotransmitter imbalance and disrupted neuronal signaling in MS rats. Another possibility for the downregulation of *Adra2a* in response to increased neurotransmitter availability could be the regulatory mechanism of desensitization. Due to the excess of NE, reduced levels of *Adra2a* could be an attempt to maintain homeostasis and prevent overstimulation of the adrenergic system. While *Adra2a* downregulation may initially be a compensatory mechanism, chronic or excessive downregulation of *Adra2a* can lead to further dysregulation of neurotransmitter systems, contributing to the development or exacerbation of depressive symptoms over time. A ligand binding activity or measurement of NE will further confirm this hypothesis. Also, it will be interesting to examine if this phenomenon is specific to ELS-induced depression.

Several studies have suggested that adrenal atrophy could be associated with prolonged exposure to stress, reflecting the organ’s adaptive response to altered physiological demands [58] and defeat in adrenal function due to hyperactivation of the HPA axis [59]. In our study, the observed reduction in adrenal gland weight suggests a possible downregulation of its function to the stress response. This finding could have significant implications for understanding the physiological stress response, and we wanted to associate that with other molecular findings. While we did not directly measure levels of adrenaline and noradrenaline in the blood or CSF, we hypothesize that reducing adrenal weight may imply lower synthesis or release of these catecholamines. In our case, the coherence observed between reduced adrenal gland weight and the transcriptional reduction of the *Adra2a* receptor in the FC suggests a potential feedback mechanism. It might also reflect a compensatory mechanism to trigger a stress-sensitive behavioral alteration.

G proteins are a family of related GTP-binding proteins which mediate the transduction of extracellular signals. G proteins are composed of α-, β-, and γ-subunits on the inner membrane surface of the cell [60]. In the inactive heterotrimeric state, GDP is bound to the Gα-subunit; however, upon activation, GDP is released, and GTP binds to Gα. Then, the combined Gα-GTP complex dissociates from Gβγ. Both Gα-GTP and Gβγ can activate downstream effectors. G_αi_ proteins contain a small subfamily of polypeptides: G_αi_1 (*Gnai1*), G_αi_2 (*Gnai2*), and G_αi_3 (*Gnai3*). The amino acid sequences of these subunits differ by only 5–15% and share overlapping functions, yet they can exhibit distinct expression patterns [61, 62]. Whereas G_αs_ stimulates adenylyl cyclase activity, G_i_ mainly inhibits adenylyl cyclase activity. In doing so, the production of cAMP from ATP decreases, and the activity of cAMP-dependent protein kinases such as PKA is also reduced. Our expression analyses of G-proteins in the intracellular signaling system downstream of the α-2A adrenergic receptor revealed interesting results. We found no change in *Gnas* mRNA transcript levels. However, the level of *Gnai2*, not *Gnai1*, was significantly decreased in the FC of MS rats. Our findings of decreased levels of the inhibitory *Gnai2* indicate less inhibition of adenylyl cyclase. As a result, one would expect elevated levels of cAMP production. In this regard, *Adra2a’s* decreased inhibitory signaling coupled with reduced expression of *Gnai2* is perplexing. The possibility that the reduction in G_αi_2-mediated signaling could trigger compensatory mechanisms that further downregulate both *Adra2a* and *Gnai2* to restore homeostasis in the face of high levels of NE cannot be ruled out. Moreover, due to the applied stress paradigm, prolonged or excessive activation of adrenergic receptors could lead to desensitization and downregulation of receptor expression as a protective mechanism to prevent overstimulation and maintain cellular homeostasis. This demonstrates that low levels of *Adra2a* and *Gnai2* are due to regulatory feedback mechanisms that aim to maintain balance in the neurotransmitter system. The reason for the selective reduction in *Gnai2* is not clear. Previous studies showed that G_αi_1 was found mainly in the brain [63, 64], while G_αi_2 and G_αi_3 were detected not only in the brain but also in peripheral tissues [65, 66]. In the developing brain, it has been shown that G_αi_1 expression was high in young rats but decreased in adults. Also, not all brain cells express G_αi_1. Further studies are needed to examine if the selective decrease in G_αi_2 in MS rats was related to their differential regulation in different cell types.

Protein phosphorylation is the key to the cAMP signaling system, primarily mediated by PKA. PKA is a tetrameric holoenzyme consisting of a regulatory (R) subunit dimer with a catalytic (C) subunit bound to each regulatory subunit. Upon binding of cAMP molecules to each regulatory subunit, PKA dissociates. This causes the release of two C subunits and an R subunit dimer. The free catalytic subunits then phosphorylate serine and threonine amino acid residues located on target substrates [67]. Two variants of PKA have been identified based on the elution profile on the diethylaminoethyl exchange column: type I and type II. These two forms differ in their structure based on the incorporated regulatory subunits termed RI or RII. Catalytic subunits can be either identical or very similar in their binding to the two types of PKA. Cloning studies show two RI subunits (RIα and RIβ), two RII subunits (RIIα and RIIβ), and three catalytic subunits (Cα, Cβ, Cγ). Each regulatory and catalytic subunit has a tissue-specific expression pattern [67]. Except for the Cγ subunit, all PKA regulatory as well as catalytic subunits are expressed in the brain. Since cAMP-mediated regulation of PKA subunits acts through gene transcription [68], mRNA stability [69], and via altered stability of the regulatory and catalytic subunits after dissociation of the holoenzyme [70], we studied the mRNA expression of all R and C subunits in the FC of MS rats. We found that the expression levels of regulatory *Prkar1β *and catalytic *Prkarcβ *were selectively reduced in MS rats. The mechanism responsible for the selectively altered expression of PKA subunits is not clear; however, there is a precedent that prolonged stimulation with cAMP can lead to a decrease in the mRNA expression of certain regulatory subunits [71]. Although our finding of lower expression G_αi_2 indicates stimulated downstream signaling, reduced levels of regulatory *Prkar1β *and catalytic *Prkarcβ *subunits, along with reduced expression of *Adra2a*, suggest overall dampened functioning of α_2_-adrenoceptors in the FC of MS rats. We and other investigators have demonstrated a similar reduced functioning of PKA in depressed subjects [33]. Given the significance of PKA in many biological functions in the brain, our observations of lower expression of *Prkar1β *and *Prkarcβ *in the FC of MS rats suggest that abnormalities in PKA may be critical in the pathophysiology of MS-induced behavioral depression.

Altogether, our present study shows an association between α_2_-adrenoceptors and subsequent signaling in ELS-induced depression. Our study opens new areas of investigation. For example, in certain regions of the brain, interactions between noradrenergic terminals and astrocytes are more common compared to those with neurons [72]. Moreover, noradrenergic neurons communicate specifically with glial cells through noradrenergic receptors expressed by glia [73, 74]. Thus, dissecting the role of α_2_-adrenoceptors and associated signaling in neurons and glia will be of great interest. Additionally, PKA cross-talks with other signaling mechanisms such as phospholipase C [75] and inositol phosphate 3 (IP3) receptors, thereby modulating Ca^2+^ influx [76] and phosphorylation of calcium-calmodulin kinase [77]. It will be worthwhile to study this crosstalk and whether PKA abnormalities co-exist with other signaling mechanisms in ELS-induced depressive behavior. Whether these abnormalities are specific to the MS paradigm or common to other types of early-life stress needs to be studied. A limitation of the study is the exclusive use of male animals, which restricts the generalizability of our findings. We plan to address this in future studies to investigate whether similar α2-adrenoceptor signaling abnormalities can be observed in female animals exposed to ELS. Additionally, our focus has been on analyzing gene expression at the transcriptional level. While gene expression data provides valuable insights into changes within the signaling system, it is critical to further examine whether these transcriptional changes correspond to alterations at the protein level. This would offer a more comprehensive understanding of how these molecular pathways influence depressive phenotypes and further validate that the observed transcriptional changes translate into functional protein modifications.

## Data Availability

No datasets were generated or analysed during the current study.
